# Valeric Acid Protects Dopaminergic Neurons by Suppressing Oxidative Stress, Neuroinflammation and Modulating Autophagy Pathways

**DOI:** 10.3390/ijms21207670

**Published:** 2020-10-16

**Authors:** Richard L. Jayaraj, Rami Beiram, Sheikh Azimullah, Nagoor Meeran MF, Shreesh K. Ojha, Abdu Adem, Fakhreya Yousuf Jalal

**Affiliations:** 1 Department of Pharmacology and Therapeutics, College of Medicine and Health Sciences, United Arab Emirates University, Al Ain 17666, UAE; richardlj@uaeu.ac.ae (R.L.J.); azim.sheikh@uaeu.ac.ae (S.A.); nagoormeeran1985@uaeu.ac.ae (N.M.M.); shreeshojha@uaeu.ac.ae (S.K.O.); fakhreya@uaeu.ac.ae (F.Y.J.); 2 College of Medicine and Health Sciences, Khalifa University, Abu Dhabi 127788, UAE

**Keywords:** Parkinson’s disease, valeric acid, rotenone, oxidative stress, neuroinflammation, neuroprotection

## Abstract

Parkinson’s disease, the second common neurodegenerative disease is clinically characterized by degeneration of dopaminergic neurons in the substantia nigra pars compacta (SNpc) with upregulation of neuroinflammatory markers and oxidative stress. Autophagy lysosome pathway (ALP) plays a major role in degradation of damaged organelles and proteins for energy balance and intracellular homeostasis. However, dysfunction of ALP results in impairment of α-synuclein clearance which hastens dopaminergic neurons loss. In this study, we wanted to understand the neuroprotective efficacy of Val in rotenone induced PD rat model. Animals received intraperitoneal injections (2.5 mg/kg) of rotenone daily followed by Val (40 mg/kg, i.p) for four weeks. Valeric acid, a straight chain alkyl carboxylic acid found naturally in *Valeriana officianilis* have been used in the treatment of neurological disorders. However, their neuroprotective efficacy has not yet been studied. In our study, we found that Val prevented rotenone induced upregulation of pro-inflammatory cytokine oxidative stress, and α-synuclein expression with subsequent increase in vital antioxidant enzymes. Moreover, Val mitigated rotenone induced hyperactivation of microglia and astrocytes. These protective mechanisms prevented rotenone induced dopaminergic neuron loss in SNpc and neuronal fibers in the striatum. Additionally, Val treatment prevented rotenone blocked mTOR-mediated p70S6K pathway as well as apoptosis. Moreover, Val prevented rotenone mediated autophagic vacuole accumulation and increased lysosomal degradation. Hence, Val could be further developed as a potential therapeutic candidate for treatment of PD.

## 1. Introduction

Parkinson’s disease (PD), the second most common neurodegenerative disease is not only characterized by motor impairments such as bradykinesia, rigidity, tremor and postural abnormalities but cognitive, emotional and olfactory abnormalities are also seen in PD patients [1,2]. Researchers believe that at least 500,000 people in the United States with a total estimated treatment cost of around $6 billion annually. Based on World population ageing report, the population of aged individuals in United Arab Emirates are expected to increase from 5.1% in 2000 to 26.7% by 2050 which creates a major concern about the incidence of PD in the UAE soon [3]. Oxidative stress is the main culprit in PD progression which in turn influences i. mitochondrial dysfunction ii. protein misfolding and iii. altered kinase activity [4]. Post-mortem studies reported that the presence of intracellular proteinaceous inclusions termed lewy bodies made up of alpha-synuclein aggregation are pathological hallmarks of PD [5]. Despite various treatment strategies such as dopamine replacement therapy, monoamine oxidase inhibitors, dopamine receptor agonists, there is limited efficiency over the course of the disease and these prolonged treatments results in adverse motor (dyskinesia, motor fluctuation) and non-motor (sleep disorder, impulse control disorder, PD dementia) side effects [6]. Various reports have recognized neuroinflammation as an important pathological mechanism driving dopaminergic neuronal loss leading to Parkinson disease [7,8,9]. Significant clinical and experimental evidence reveal that microglia and astroglia mediated neuroinflammation plays a major role in neurodegeneration [10,11]. Microglia activation is portrayed by enhanced expression of pro-inflammatory factors (ROS, TNF-α, IL-1β, COX-2, iNOS), rapid microglial population expansion and morphological transformation [12,13]. PET imaging studies have proved widespread microglial response in PD patients with subsequent augmentation of oxidative and nitrosative stress [14,15], fueling inflammation mediated neurodegeneration, Astrocytes which represents 50% of glial population are also being studied for its significant role in initiation of PD. Moreover, it has been reported that astrocytic alpha-synuclein deposits trigger recruitment of activated microglia that executes neurons resulting in PD [16,17]. Though astrocytes exert neuroprotective role in normal physiological condition, during diseased state, reactive astrogliosis aid in release of inflammatory cytokines that damage surrounding neurons by production of ROS, lipid peroxidation and activation of apoptotic mechanisms [18,19]. Kim et al., proved that dopaminergic neurons treated with MPP^+^ released MMP3 which are responsible for TNF-α and ROS production by microglia [20,21]. Although various pathways initiates toxic events leading to neurodegeneration, the most common cause of PD pathogenesis is due to abundant deposition of misfolded proteins (α-synuclein) and impairment of protein degradation pathways leading to neuronal death. One such important protein degradation mechanism is called autophagy. Alternate to ubiquitin-proteosome system which degrades 80–90% of short lived proteins, macroautophagy plays a major role in degradation of non-specific long-lived proteins as well as cellular organelles. Autophagy is an intracellular catabolic process in which misfolded proteins such as α-synuclein are isolated by autophagosomes which eventually fuses with endosomes to form amphisomes. The contents in the amphisomes are digested and recycled when amphisomes fuses with lysosomes [22,23]. However, studies have reported that impairment of autophagy leads to accumulation of pathogenic α-synuclein in PD [24,25]. In addition, over-expression of mutant α-synuclein have been shown to activate autophagy [26]. Impairment of mammalian target of rapamycin (mTOR), an vital protein for cell proliferation, growth and survival [27,28] leads to dopaminergic neuronal loss. Similarly, downregulation of mTOR using siRNA interference hinders phosphorylation of both p70 S6 Kinase (p70S6K) and eukaryotic initiation factor 4E binding protein 1 (4E-BP1) which results in neuronal apoptosis [29]. In addition, PD mimetics such as 6-hydroxydopamine (6-OHDA), N-methyl-4-phenylpridine (MPP^+^) and rotenone abolished mTOR signaling and up-regulated cleaved caspase-3 leading to neuronal apoptosis [30]. Interestingly, restoration of mTOR, 4E-BP1 or p70S6K significantly protected neurons against PD mimetics [31,32].

Rotenone, a mitochondrial complex I inhibitor is one of the most widely used neurotoxin to study the biochemical and molecular changes reminiscent to pathological changes occurring in PD. Unlike MPP^+^ which required transporters to induce dopaminergic neuronal death, rotenone is highly lipophilic and thus eventually crosses blood-brain barrier effectively, Various studies have shown that oxidative stress and impairment of mitochondrial function results in accumulation of autophagy vacuoles and toxicity [33,34]. In addition to deterioration of normal autophagy process, rotenone have also been known to cause neuroinflammation, apoptosis of dopaminergic neurons and behavioral deficits [30]. Hence, to counteract these toxic oxidative stress mediated dopaminergic neurodegeneration, natural drugs with robust anti-oxidant and anti-inflammatory properties that not only helps to restore normal autophagy process but also when it guards dopaminergic neurons and alleviate disease progression.

Valeric acid (Val) or pentanoic acid, a prime component of *Valeriana wallchii* DC., Caprifioliaceae has potent biological activities with a molecular weight of 102.15 and chemical formula C_5_H_10_O_2_ has potent biological properties. *Valeriana* has extensive traditional reputation for its protective activity against insomnia, neurosis, pain [35,36] and depression [37,38]. Moreover, Val has an identical structure to neurotransmitter gamma-aminobutyric acid (GABA) but without amine functional group which is responsible for GABA’s biological activity. Further, Val is also an N-Methyl-D-aspartate (NMDA) antagonist and protects against penicillin-induced epileptic activity in rats [39]. Nevertheless, to the best of our knowledge, there were no studies reporting the neuroprotective mechanisms of Val in rotenone induced PD model. Hence, in this study, using a chronic intraperitoneal rotenone treatment as an in vivo PD model, we examined the neuroprotective efficacy of Val against rotenone induced oxidative stress, neuroinflammation, α-synuclein expression and dopaminergic neuronal loss. In addition, we evaluated whether the probable mechanisms of Val mediated protection is associated with mTOR-arbitrated anti-apoptotic and/or autophagic events.

## 2. Results

### 2.1. Valeric Acid Prevented Rotenone Induced Oxidative Stress in Rats

**Figure 1:**
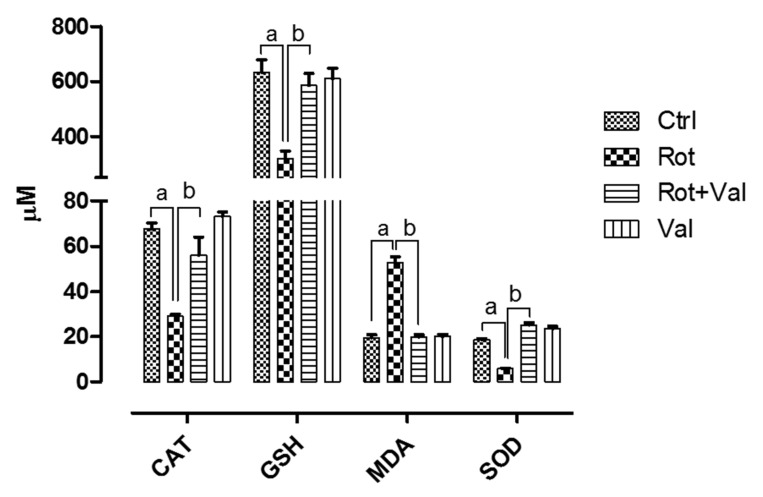
Influence of Valeric acid on rotenone induced oxidative stress and antioxidative indices rats. Rotenone administration caused a significant increase (*p* < 0.05) in lipid peroxidation which is represented by increase in malonaldehyde levels with marked decrease in vital anti-oxidants levels in the midbrain. Treatment with 40 mg/kg Valeric acid prevented lipid peroxidation with a significant decrease in MDA levels and ameliorated brains vital antioxidant (CAT, GSH and SOD) levels. There is no significant difference in both MDA and antioxidant levels in control and Val alone treated groups. Data are expressed as mean ± SEM. ^a^ *p* < 0.05 compared to control, ^b^ *p* < 0.05 compared to rotenone treated group.

The dosage of Val used in this study did not show any toxicity and it was selected based on previous study [40]. Antioxidant enzymes plays a major role in scavenging reactive oxygen species which is a product of oxidative metabolism. Four weeks after rotenone administration, we found an robust decrease in vital antioxidants (Catalase, Glutathione and Superoxide dismutase) and significant increase in malondialdehyde (MDA) levels, an marker of lipid peroxidation. However, oral administration of Val after rotenone exposure significantly increased antioxidant levels with concomitant decrease in MDA (Figure 1). These results demonstrate that Val can scavenge rotenone-induced oxidative stress in rats.

### 2.2. Valeric Acid Ameliorated Rotenone Induced Neuroinflammation

**Figure 2:**
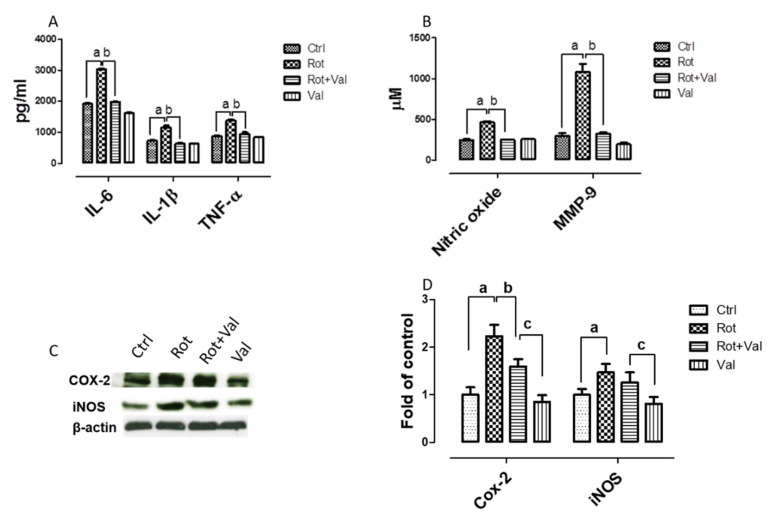
Valeric acid prevented nitric oxide production and alters expression of inflammatory factors in rotenone treated animals. Enzyme linked Immunosorbent assay showed that rotenone administration increased the expression of pro-inflammatory cytokines (**A**) and enhanced production of NO and MMP-9 (**B**). Immunoblots of midbrain protein samples probed with Cox-2 and iNOS (**C**). Blots were quantified using Image J and corresponding results were represented as bar diagram (**D**). However, Val treatment caused a significant decrease in expression and production of pro-inflammatory factors in rotenone intoxicated animals. Data are expressed as mean ± SEM. ^a^ *p* < 0.05 compared to control, ^b^ *p* < 0.05 compared to rotenone treated group, ^c^ *p* < 0.05 compared to Rot+ Val treated group.

We next examined the ability of Val to modulate the production and secretion of inflammatory factors in rotenone induced PD model. It is well established that pro-inflammatory cytokines augment neurodegenerative mechanisms. Our results showed that rotenone injection induced a significant increase in production of pro-inflammatory factors which is evident from increase in interleukin-6 (IL-6), interleukin-1β (IL-1β), tumor necrosis factor-α (TNF-α), nitric oxide (NO) and matrix metalloproteinase 9 (MMP-9) (Figure 2A,B). Notably, administration of Val potentially diminished the production of pro-inflammatory factors thereby establishing Valeric acid mediated anti-inflammatory mechanism. In addition, enhanced expression of inducible nitric oxide synthase (iNOS) and cyclooxygenase 2 (COX-2) have been reported in the brain of PD patients and also causes dopaminergic neuronal loss in PD models [11,41]. Consistent with previous reports, rotenone administration caused a significant increase in expression of iNOS and COX-2, whereas Val treatment markedly reduced these protein levels as evinced by western blotting (Figure 2C,D).

### 2.3. Valeric Acid Diminished the Activation of Microglia and Astrocytes

**Figure 3:**
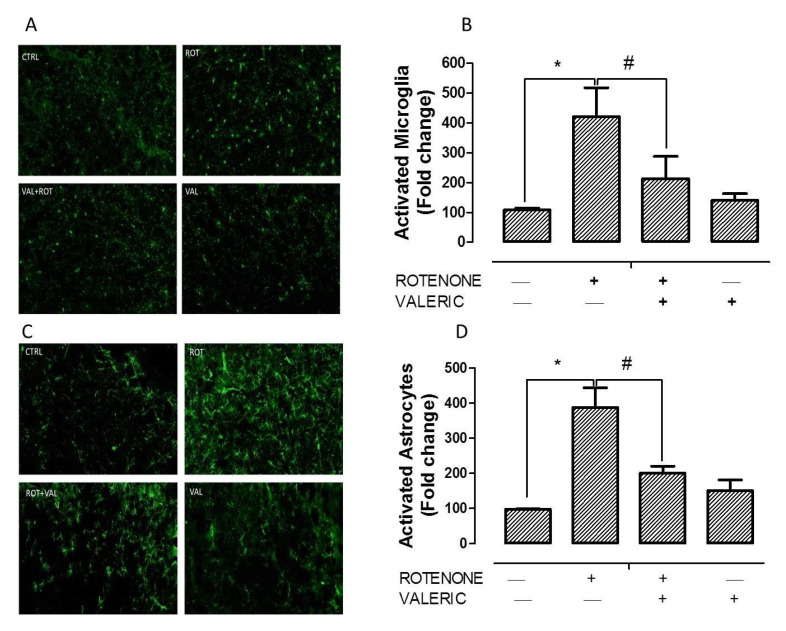
Valeric acid attenuated microglia and astrocyte activation induced by rotenone. Immunofluorescent staining with Iba-1 (**A**) and GFAP (**C**) of experimental animals. Picture shows 20 µm thick sections of striatum between different groups. Densitometric assessment of fluorescent intensity to quantify microglia (**B**) and astrocyte (**D**) activation. Rotenone administration caused profound increase in Iba-1 positive microglia and GFAP positive astrocyte. Alternatively, Val treatment decreased the activation of microglia and astrocyte as evinced by decrease in Iba-1 and GFAP positive cells. Values are expressed as mean ± SEM. * *p* < 0.05 compared to control, ^#^ *p* < 0.05 compared to rotenone treated group.

Enhanced expression of glial markers, especially ionized calcium-binding adapter molecule 1 (Iba-1) and glial fibrillary acidic protein (GFAP), is an indicator of reactive microgliosis and reactive astrocytes respectively. These two cells play a major role in inflammation mediated neurodegeneration in PD. Figure 3 shows that immunofluorescent staining for Iba-1 and GFAP activated microglia and astrocytes respectively in the striatum of experimental animals. Activation of microglia by rotenone is evident from larger cell bodies and fewer processes (Figure 3A). Similarly, astrocyte activation by rotenone is denoted by enhanced expression of GFAP positive cells (Figure 3C). Interestingly, administration of Val in rotenone treated rats significantly reduced the expression of Iba-1 and GFAP in the striatum of experimental animals. These results were in concordant with the diminished production of pro-inflammatory factor production described previously. Quantification of activated microglia and astrocyte are represented as percentage of control and depicted as histogram in Figure 3B,D respectively.

### 2.4. Valeric Acid Cosseted Dopaminergic Neurons against Rotenone Toxicity

**Figure 4:**
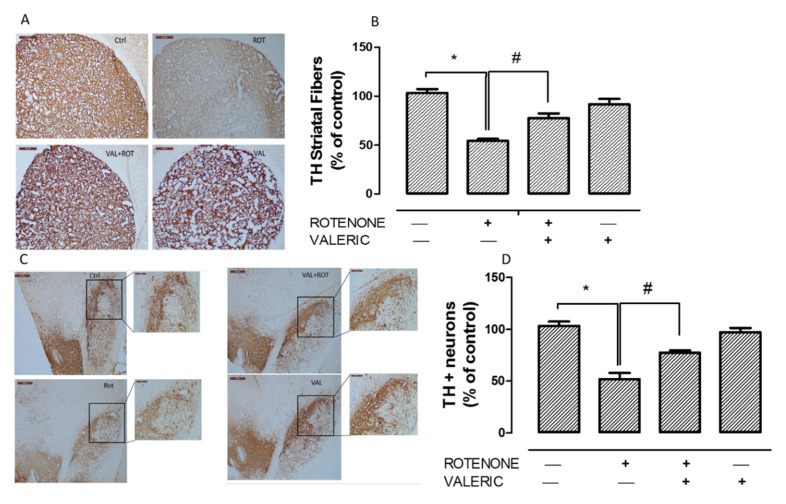
Valeric acid attenuated rotenone induced dopaminergic neurodegeneration. Immunohistochemistry of 20 µm coronal sections with anti-tyrosine hydrolase antibody of striatum (**A**) and substantia nigra (**B**) of experimental animals. Densitometric analysis of tyrosine hydrolase positive neuronal fibers (**C**) and tyrosine hydrolase positive neurons (**D**) were performed using Image J. Administration of rotenone cause a significant reduction in both TH+ve neuronal fibers and neurons in striatum and substantia nigra respectively. Whereas, administration of Val prevented this loss significantly. Values are expressed as mean ± SEM. * *p* < 0.05 compared to control, ^#^ *p* < 0.05 compared to rotenone treated group.

Loss of tyrosine hydrolase (TH) positive neurons in the substantia nigra pars compacta (SNpc) and the resultant decrease of TH expression in the striatum are hallmark pathological events in PD. Hence, we measured TH+ve dopaminergic neurons in SNpc and its expression in the striatum. Administration of rotenone for 4 weeks, caused a significant reduction in the number of TH positive neurons (Figure 4A,B) which in turn caused 50% decrease in the intensity of TH+ve striatal fibers (Figure 4C,D). Significantly, we found that administration of Val to rotenone toxicated rats prevented dopaminergic neuronal loss and enhanced TH expression in striatal fibers as demonstrated by immunohistochemistry.

### 2.5. Valeric Acid Prevented α-Synuclein Aggregation in PD Rats

**Figure 5:**
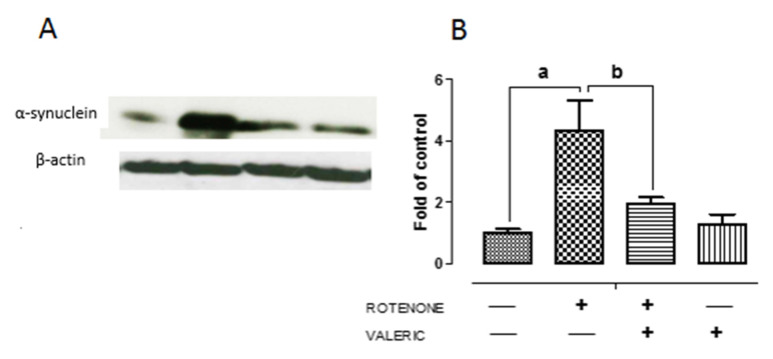
Valeric acid treatment regulates the expression of α-synuclein in experimental animals. Representative immunoblots of alpha-synuclein expression in experimental animals (**A**). Band intensity was quantified using Image J and depicted as bar diagram (**B**). Data are expressed as mean ± SEM. ^a^ *p* < 0.05 compared to control, ^b^ *p* < 0.05 compared to rotenone treated group.

Accumulation of α-synuclein in Lewy bodies is an key pathological event in PD and enhanced expression of α-synuclein influences neuronal death by either necrosis or apoptosis [42]. Moreover, impairment of autophagy results in accumulation of aggregated α-synuclein protein favoring neurodegeneration. We found that rotenone administration caused a significant increase (3 fold) in α-synuclein protein levels in the substantia nigra pars compacta as shown by western blotting (Figure 5). However, we noted that treatment with Val caused a significant decrease in α-synuclein expression. These results indicate Val partly protects dopaminergic neurons by diminishing the expression of α-synuclein in rotenone treated animals.

### 2.6. Valeric Acid Hindered Neuronal Apoptosis by Reinstating mTOR Pathway

**Figure 6:**
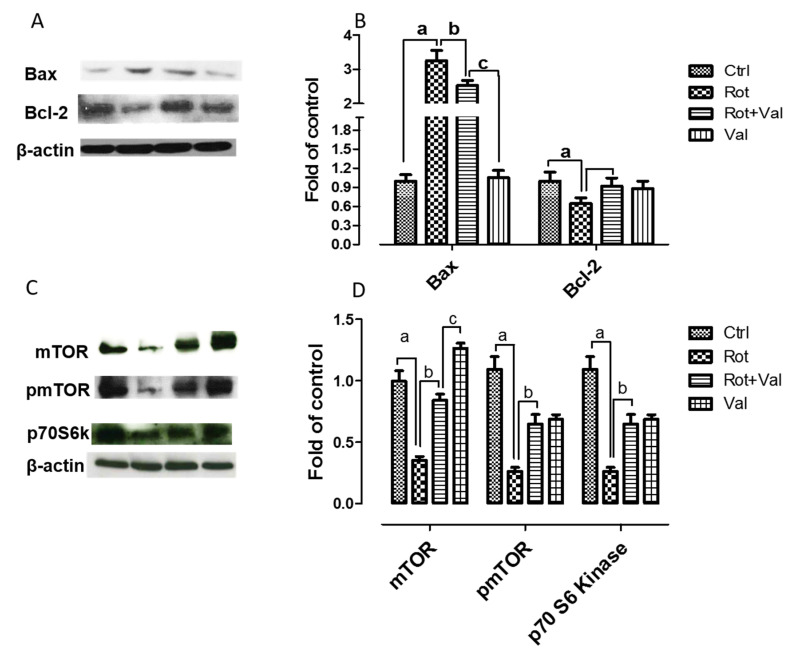
Valeric acid prevented rotenone mediated neuronal apoptosis by restoring mTOR pathway. Immunoblotting of midbrain samples with mTOR pathway (**A**) proteins and apoptotic markers (**C**). Blots for phosphor mTOR and p70S6K (**B**), Bax and Bcl-2 (**D**) mTOR were quantified using Image J and represented as fold of control. Values are expressed as mean ± SEM. ^a^ *p* < 0.05 compared to control, ^b^ *p* < 0.05 compared to rotenone treated group, ^c^ *p* < 0.05 compared to Rot+Val treated group.

Various reports have shown that impairment of mTOR activity leads to neuronal dysfunction and have adverse effect on neuronal regenerative mechanisms [43,44]. p70 S6 kinase (p70S6K) is one of the most defined downstream effector molecules of mTOR. In central nervous system, aggregated protein toxicity initially increases mTOR activity but its expression is subsequently reduced leading to cell death [45]. In this study, we wanted to evaluate if Val treatment could prevent neuronal apoptosis by reinstating mTOR activity. Immunoblotting analysis showed that rotenone administration caused a significant decrease in mTOR, phospho mTOR and p70S6K expression (Figure 6A,B). Whereas, Val administration restored mTOR activity by enhancing the expression of these proteins. In addition, to evaluate the importance of mTOR activity on neuronal survival and to assess if Val could regulate apoptosis signaling pathway, we analysed the levels of Bax and Bcl-2 by western blotting. We found that rotenone administration caused a significant increase in the expression of pro-apoptotic protein Bax and decreased the expression of anti-apoptotic protein Bcl-2. By contrast, Val administration prevented apoptosis by diminishing the expression of Bax and enhancing Bcl-2 expression (Figure 6C,D). Together, these results suggest that Val prevented rotenone mediated apoptosis by reinstating mTOR pathway.

### 2.7. Valeric Acid Prevented Rotenone Toxicity via Regulating Autophagy

**Figure 7:**
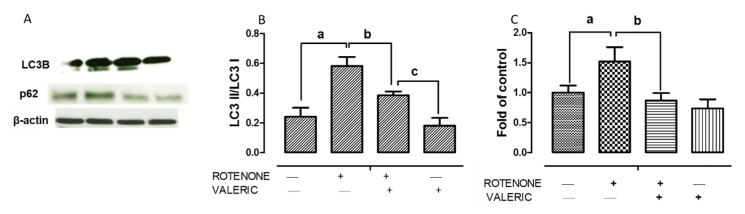
Valeric acid cosseted dopaminergic neurons by modulating autophagy. Immunoblotting of midbrain samples with autophagy markers LC3 II/LC3 I and p62 (**A**) from different groups. Quantitative analysis of immunoblotting of LC3-II/LC3-I (**B**) and P62 (**C**) both controlled by β-actin, respectively. Values are expressed as mean ± SEM. ^a^ *p* < 0.05 compared to control, ^b^ *p* < 0.05 compared to rotenone treated group, ^c^ *p* < 0.05 compared to Rot+Val treated group.

As mentioned earlier, accumulation of misfolded α-synuclein due to impairment of autophagy plays a central role in lewy body formation and resultant neurodegeneration in PD. Hence, to further delineate neuroprotection mechanisms of Val, we analyzed whether Val could modulate rotenone impaired autophagy using MAP-light chain 3 (LC3) and p62 protein expression levels. Treatment with rotenone caused a significant increase in autophagosome accumulation which is demonstrated by enhanced LC3II/LC3I ratio. Whereas, Val administration to rotenone intoxicated rats significantly reduced LC3II/LC3I ratio demonstrating decrease in autophagosome accumulation. (Figure 7A,B). To further assess the role of Val in autophagy regulation, we assessed the expression of p62 which helps to link ubiquitinated proteins to autophagic process through LC3. Administration of rotenone caused a significant increase in p62 levels demonstrating that rotenone inhibits autophagic degradation. Alternatively, treatment with Val caused a significant decrease in p62 expression levels suggesting that it favors autophagic degradation of misfolded proteins (Figure 7C,D). In addition, the ratio of LC3II/LC3I and p62 levels did not significantly increase in both control and Val alone treated groups.

## 3. Discussion

In this present study, we evaluated the neuroprotective effect of Val using rotenone induced PD rat model. As of our knowledge, this is the first study to report that the neuroprotective mechanisms of Val involve attenuation of oxidative stress, reduction of pro-inflammatory factors by modulating microglia and astrocyte response and down regulation of α-synuclein expression. Moreover, Val protected dopaminergic neurons by restoring rotenone inhibited mTOR and autophagy pathways.

Rotenone, an natural insecticide from *Leguminosa* plants is a prototypical neurotoxin that can mimic pathological features of PD. Rotenone is an potent mitochondrial complex I inhibitor that easily crosses blood brain barrier without the need of dopamine transporter for dopaminergic neuron entry. It is well established in our lab and by others that rotenone exposure causes pathological features of PD such as oxidative stress, nigrostriatal neuron loss, behavior deficits, inflammation, cytoplasmic α-synuclein inclusions, defective protein turnover and autophagy [46,47,48]. Mounting evidence shows that brains of PD patients have significant depletion of antioxidant enzymes [49], impairment of complex I activity of mitochondrial electron transport chain [50], increased protein oxidation/nitration and iron levels [51,52]. Based on similar reports, it is well accepted that redox imbalance is not a secondary end-stage factor in PD but they are the main culprits driving PD progression. Hence, any drug that has the ability to effectively scavenge or inhibit reactive oxygen species (ROS) could be a potential therapeutic agent for PD. Valeric acid, an terpenoid ester from *Valeriana* species have been known to possess various biological activities [35]. *Valeriana* has got extensive reputation for its ability to treat pain insomnia, epilepsy, neurosis, anxiety and depression [36,37,38]. Though *Valeriana* and its bioactive component Val possess various biological properties, its antioxidant ability has not been studied previously. In this study, similar to previous reports, rotenone administration caused a significant decrease in vital antioxidants GSH, CAT and SOD with a strong increase in MDA, a marker for lipid peroxidation. However, treatment with Val significantly reduced rotenone mediated oxidative stress by increasing vital antioxidant enzymes with a significant decrease in lipid peroxidation.

Various studies have demonstrated that oxidative stress and neuroinflammation are co-conspirators in PD progression. Over the past few years, various clinical reports provide experimental evidence that inflammation could aggravate neurodegeneration in PD with similar observations in both toxin and genetic models [53]. Generally, oxidants are reactive factors that influence neuronal death but it is interesting to note that in an MPTP based PD model, the time course of oxidative stress did not correlate with neurodegeneration. Moreover, oxidative alternation of α-synuclein [54], tyrosine hydrolase [55] precede dopaminergic neurodegeneration. Hence it may be plausible that oxidants rather than directly killing neurons, they act as key signaling molecules activating neuroinflammation [49], impairment of autophagy systems and apoptosis of dopaminergic neurons [56]. In the central nervous system, microglia acts as a major source of reactive oxygen species through oxidative mechanisms in mitochondria and intracellular peroxidases [57]. Similar to previous reports, we found that rotenone mediated increase in oxidative stress amplifies activation of microglia and astrocyte [58]. In PD, it is believed that dopaminergic neurons die overtime by nonsynchronous events [59] and this toxic modification in brains microenvironment, though subtle, can be immediately perceived by microglia and astrocyte. Thus, activated microglia and astrocyte produce enhanced pro-inflammatory factors such as IL-6, IL-1β, TNF-α and NO. This increase have also been observed in post-mortem brain and CSF of PD patients [60,61,62]. Moreover, Duke et al., 2007 reported an increase in expression of genes encoding pro-inflammatory cytokines in substantia nigra of PD patients [63]. In line with these reports, we found that rotenone administration caused hyper-activation of microglia and astrocyte (Figure 3) which inturn enhanced the expression of IL-6, IL-1β, TNF-α, NO, MMP-9, COX-2 and iNOS (Figure 2). However, treatment with Val diminished microglia and astrocyte activation with subsequent reduction in pro-inflammatory factors.

One of the important factor to enhance neuroinflammation, apart from neurodegeneration is enhanced expression of α-synuclein. Watson et al., found that over-expression of α-synuclein caused microglia activation as well as production of pro-inflammatory factors [64]. In our study, we found that Val administration abolished rotenone mediated increase in α-synuclein expression (Figure 5). Thus, in our study, decline in α-synuclein could partly inhibit activation of pro-inflammatory factors. Our reports were similar to previous studies were rotenone administration caused α-synuclein positive nigral inclusions and increase in misfolded/dysfunctional protein that facilitated dopaminergic neuronal death [65]. Further we evaluated whether anti-oxidative and anti-inflammatory mechanism of Val could protect nigrostriatal dopaminergic neurons. We found that administration of rotenone caused a significant degeneration of dopaminergic neurons in SN and decrease in tyrosine hydrolase expression in striatal fibers (Figure 4). This results were in concordant with our previous report [46]. However, administration of Val prevented both TH positive dopaminergic neuronal loss and TH positive striatal fiber loss in rotenone treated animals. Valeric acid mediated suppression of pro-inflammatory mechanisms and protection of dopaminergic neurons can be arbitrated by various mechanisms.

Apoptosis, an well-conserved evolutionary process plays a major role in dopaminergic neurodegeneration. Although various reports prove the presence of apoptotic neurons in substantia nigra of PD patients [66,67], Dispasquale et al., 1991 was the first to report that MPP^+^, an mitochondrial complex I inhibitor induced apoptosis in cerebellar granule neurons [68]. Moreover, expression of wild type or mutant forms of α-synuclein causes neuronal apoptosis and also augments neuronal sensitivity to apoptotic death [69]. Although two major pathways leads to apoptosis (intrinsic and extrinsic), intrinsic pathway is mostly activated by ROS, DNA damage, loss of trophic support. The central phenomenon in intrinsic apoptotic pathway is the release of cytochrome c into the cytosol, membrane permeabilization by pore-forming protein Bax, decrease in anti-apoptotic protein Bcl-2 that results in diminished ATP synthesis, enhanced ROS production, swelling of mitochondria and ultimately cell death. Also, high concentration of α-synuclein down-regulates the expression of anti-apoptotic protein Bcl-2 [70]. Zhou et al., 2015 recently showed that inhibition of mTOR signaling leads to rotenone mediated neuronal apoptosis [32]. The mTOR cascade plays a major role in cell shape, migration and differentiation during neuronal development [71] and they have been found to be essential for memory formation [72,73] and synaptic plasticity [32], Moreover, inhibition of mTOR arbitrated p70S6K and 4E-BP1 promotes apoptosis [27]. Rotenone administration promotes neuronal apoptosis by inhibiting mTOR cascade suggesting the importance of mTOR pathway in neuroprotection [32]. Similar to these reports, we found that rotenone administration decreased the expression of mTOR, phospho mTOR and p70S6K and provoked neuronal apoptosis by increasing the expression of Bax and decreasing Bcl-2 expression. However, administration of Val to rotenone intoxicated animals restored mTOR pathway by increasing the expression of mTOR, phospho mTOR and prevented neuronal apoptosis. Hence, our study suggest that Val prevented rotenone mediated inhibition of mTOR signaling and also rescued neurons from apoptosis.

Numerous clinical and in vitro studies have provided strong evidence that accumulation of autophagic vacuoles is detrimental to dopaminergic neurons [34,67,74]. During normal physiological conditions, α-synuclein will be degraded by both autophagy-lysosome pathway (ALP) and proteasome system but during α-synuclein overload (in PD), misfolded or aggregated α-synuclein will be eliminated by ALP predominantly [75]. Though autophagy remains primarily as a protective mechanism to maintain nutrient and energy homeostasis during stress, impairment of autophagy results in PD [76]. Mader et al., 2012 demonstrated that rotenone administration induced oxidative stress, α-synuclein accumulation, dopaminergic neuron death along with accumulation of autophagic vacuoles indicated by enhanced LC3 expression). Moreover, accumulation of autophagic vacuoles were correlated with decrease in lysosomal degradation which was corroborated with increase in p62 levels (autophagy substrate) [77]. Similar to this study, we found that rotenone administration caused a significant increase in LC3II/LC3I ratio supporting the accumulation of autophagic vacuole. However, administration of Val resulted in significant decrease in LC3II/LC3I ratio levels. Since, accumulation of autophagic vacuole might be due to autophagy induction or due to decline in lysosomal degradation, autophagic flux is usually assessed by detecting p62 levels, an ubiquitinating and LC3 binding protein that attaches with misfolded proteins resulting to autophagy degradation. However, rotenone administration increases p62 levels demonstrating that rotenone disrupts autophagic vacuole degradation [77]. Alternatively, Val administration caused a significant decrease in p62 levels. To conclude, in concordant with previous report, rotenone accumulates autophagic vacuoles and impairs autophagic flux [77]. Moreover, mTOR is an negative regulator of autophagy [78]. These data suggest that Val confers neuroprotection by decreasing the expression of α-synuclein and activating mTOR pathway that results in less autophagic vacuole formation and inhibited autophagy flux impairment.

## 4. Materials and Methods

### 4.1. Experimental Animals and Ethics Statement

All experimental animal procedures were approved by Animal Ethics Committee of United Arab Emirates University (UAEU). Adult male Wistar rats (6–7 months old) weighing 280–300 g were used in this study. Animals were maintained at pathogen free facility with ambient conditions (22 ± 1 °C, 60% humidity, and 12 h diurnal cycle) and had ad libitum access to food and water. Animals were allowed 1 week initially to adapt to the experimental room conditions.

### 4.2. Chemicals and Reagents

Rotenone, Valeric acid, RIPA lysis buffer, antibodies against inducible nitric oxide synthase (iNOS), cyclooxygenase-2 (Cox-2) and glial fibrillary acidic protein (GFAP) were procured from Sigma-Aldrich, St. Louis, MO, USA. Protease and phosphatase inhibitor cocktail were procured from Thermo Scientific, USA. Anti-tyrosine hydrolase (Polyclonal rabbit) antibody was obtained from Merck, Germany. The following antibodies were purchased from Cell Signalling Technology, Beverly, MA, USA: LC3, p62, mTOR, phosphor mTOR and p70S6K. Apoptotic polyclonal markers (Bax and Bcl-2) were obtained from Abcam, USA. Monoclonal mouse anti-α-synuclein antibody was purchased from BD Biosciences, San Jose, CA, USA. Anti- Iba-1 antibody was purchased from Wako chemicals, Richmond, VA, USA. Fluorescent secondary antibodies (Alexa Flour 488) were purchased from Thermo Fischer Scientific, Waltham, MA, USA. Biotinylated goat anti-rabbit secondary antibody was purchased from Jackson Immunoresearch, West grove, PA, USA. Biochemical assays were performed using commercially available kits. All other chemicals used in this experiments were provided by local commercial sources (analytical grade quality).

### 4.3. Experimental Design

To analyze the neuroprotective effect of Val, we used an established chronic rotenone paradigm which represents one of the most stable toxin based PD models. Rotenone (2.5 mg/kg) preparation were described in detail previously [46]. Pharmacological effects of Val in vivo were examined using the following treatment groups (*n* = 15). Group I (control) received intraperitoneal injection of myglol and olive oil which are vehicles for rotenone and Val respectively which served as control group. Group II (rotenone) received intraperitoneal injection of rotenone (2.5 mg/kg) once daily for 4 weeks and served as experimental PD model. Group III (rotenone+ Val): Immediately before treatment, Val (40 mg/kg, i.p) was prepared and administered once daily for four weeks, 30 min prior to ROT administration. Dosage fixed for Val was based on previous study [40]. Group IV received only intraperitoneal injection of Val (40 mg/kg, i.p) once daily for four weeks and served as drug control. Body weight of experimental animals were obtained every 5 days for a total period of 4 weeks.

### 4.4. Tissue Processing

At the end of the experiments, animals were anesthetized using pentobarbital (40 mg/kg body weight) and perfused via intracardial infusion with 0.01 M phosphate-buffered saline at pH 7.4. For immunohistochemical studies, after infusion with 0.01 M PBS, animals were again perfused with 4% paraformaldehyde for whole body fixation. Brains were then removed from the skull and post-fixed again in 4% paraformaldehyde for 48 h at 4 °C followed by cryoprotection using 30% sucrose solution for three consecutive days (4 °C). For biochemical studies, midbrain and striatum were dissected on dry ice and immediately homogenized using KCl buffer (Tris–HCl 10 mM, NaCl 140 mM, KCl 300 mM, ethylenediaminetetraacetic acid 1 mM, Triton-X 100 0.5%) at pH 8.0 supplemented with protease and phosphatase inhibitor. The homogenates were centrifuged at 14,000× *g* for 20 min (4 °C) and the supernatant was used for evaluation of lipid peroxidation, antioxidant enzymes and proinflammatory cytokines using spectrophotometric measurements and enzyme-linked immunosorbent assays (ELISA).

### 4.5. Malondialdehyde Assay

This assay was performed to assess the extent of lipid peroxidation in experimental animals using North West Life Science (Vancouver, WA, USA) MDA detection kit. Briefly, 250 μL of samples or calibrators were incubated with thiobarbituric acid followed by rigorous vortexing. After 1 h incubation at 60 °C, the mixture were centrifuged at 10,000× *g* for 2–3 min and the reaction mixture was transferred to cuvette. Spectra was measured at 532 nm and the results were expressed as μm MDA/mg protein.

### 4.6. Quantification of Reduced Glutathione

Levels of reduced glutathione in tissue homogenates were measured as previously described [46] using Sigma’s glutathione assay kit (Sigma-Aldrich Chemie GmbH, Steinheim). In brief, samples were first deproteinized with 5% 5-sulfosalicylic acid solution, centrifuged to remove the precipitated protein and supernatant was used to estimate GSH. Ten microlitre samples or standards were incubated with 150 μL of working mixture (assay buffer +5,5′-dithiobis (2-nitrobenzoic acid) + GSH reductase) in 96-well plates for 5 min. Diluted NADPH solution (50 µl) was added into each well and mixed thoroughly. Absorbance was measured at 412 nm with the kinetics for 5 min by using the microplate reader. Results were expressed as μm GSH/mg protein.

### 4.7. Assay for Antioxidant Enzyme Activities

Cayman assay kits (Cayman Chemicals Company, Ann Arbor, MI, USA) were used to assess antioxidant enzyme [superoxide dismutase (SOD) and catalase (CAT)] activities in experimental animals. Catalase assay: Twenty microliter of samples or standards and 30 μL of methanol was added to the assay buffer (100 μL) in 96-well plates. To this mixture, twenty microliter of hydrogen peroxide was added and incubated for 20 min at room temperature (RT) to initiate the reaction. Following incubation, 30 μL of Potassium hydroxide was used to terminate the reaction, followed by subsequent addition of catalase purpald (30 μL) and catalase potassium periodate (10 μL). The plate was incubated for 5 min at room temperature in a shaker and the plate was read at 540 nm using microplate reader. Catalase activity was expressed as nmol/min/mg protein. Superoxide dismutase assay: Ten microliters of samples or standards were added in 96-well plates. Twenty microliter of xanthine oxidase was added to initiate the reaction. The reaction mixture was mixed for few seconds and incubated (covered) for 30 min at RT. Absorbance was read at 450 nm using microplate reader. The activity of SOD was expressed as units/mg protein.

### 4.8. Estimation of Nitrite Levels

Nitrite levels in experimental animals were assessed using commercially available R&D Nitrite kit (Minneapolis, MN, USA). Briefly, nitrite standard or samples (50 µL) were added to 50 µL of reaction diluent in a 96-well plate (supplied along with the kit). Fifty microliters of Griess reagent was then added into each well and the reaction mixture in the plate was mixed by gentle tapping. The plate was incubated at room temperature for ten minutes and absorbance was read at 540 nm. Nitrite levels were expressed as µmol/mg protein.

### 4.9. Proinflammatory Cytokines and MMP-9 ELISA Assay

To analyze the effect of Val on rotenone induced pro-inflammatory cytokines (TNF-α, IL-1β, IL-6) and MMP-9, we used commercially available ELISA kits (BioSource International Inc., Camarillo, CA, USA). Briefly, 96 well plates were coated with 100 μL of capture antibody (diluted) and incubated overnight at room temperature. Following incubation, each well was washed using wash buffer (0.05% Tween 20 in PBS 0.01 M pH 7.4) and blocked with blocking buffer [1% bovine serum albumin in PBS (300 μL)] for 1 h. The plates were then washed with washing buffer and standards or samples (100 μL) were added into each well and incubated for 2 h at RT. Hundred microliter of detection antibody was then added into each well and the plate was incubated at room temperature for 2 h. Following incubation, the plates were washed and 100 μL of working solution (1:200, streptavidin horseradish peroxidase) was added and the plate was incubated for 20 min. The wells were then exchanged with 100 μL substrate solution and the plate was again incubated further for 20 min. Finally, stop solution [2N H_2_SO_4_, (50 μL)] was added into each well and the contents in the plate were mixed plate by gentle tapping. The plate was read immediately at 450 nm using microplate reader. The results were expressed as pg/mg protein.

### 4.10. Assessment of Microglia and Astrocyte Activation by Immunofluorescence Staining

Immunofluorescence staining was performed to analyze the anti-inflammatory effect of Val in experimental animals. Twenty micrometer thick striatum sections were washed twice with PBS and incubated for 1 h with blocking reagent (10% normal goat serum in PBS 0.3% Triton-X 100) at room temperature. The sections were then incubated with anti-rabbit Iba-1 (1:1000) and anti-rabbit GFAP (1:1000) antibodies for overnight at 4 °C. After incubation, the sections were washed twice with PBS and incubated with corresponding fluorescent secondary antibody (Alexa 488 anti-rabbit) for 1 h at RT. The stained sections were again washed twice with PBS and mounted using Vectastain fluorescent mounting media (with DAPI). The images were taken under Nikon Eclipse Ni fluorescent microscope.

### 4.11. Quantification of Activated Astrocytes and Microglia in the Striatum

A minimum of three coronal sections of the similar level of striatum from each brain were used to analyze activated astrocyte and microglia. Activated astrocytes and microglia were assessed using previously published method [79]. Three different fields of equal area were randomly chosen and analyzed using the Image J software (NIH, Bethesda, MD, USA). Briefly, an outline was drawn around the region of interest and area, circularity, mean fluorescence was measured, along with several adjacent background readings. The total corrected cellular fluorescence (TCCF) was calculated using the formula, TCCF = integrated density–(area of selected cell × mean fluorescence of background readings). All readings were measured by an observer blind to the treatment conditions to guarantee the lack of bias. Results were represented as percentage of control.

### 4.12. Immunoblot Analysis

The expression of α-synuclein, COX-2, iNOS, Bax, Bcl-2, mTOR, phospho mTOR, p62, LC3 and p70S6K in striatum of experimental animals were assessed using previously published western blotting protocol [46]. In brief, tissues were homogenized in RIPA buffer supplemented with protease and phosphatase inhibitor and centrifuged at 12,000 rpm for 20 min. Equal amount of protein (20 µg) from each sample were loaded and separated on SDS-PAGE. The separated proteins were then electrotransferred onto a PVDF membrane by semi-dry transfer method (BIO-RAD). After blocking (1 h in 5% nonfat dry milk in TBS at RT), the membranes were incubated with α-synuclein (1:750), COX-2 (1:2000), iNOS (1:1000), Bax (1:2000), Bcl-2 (1:500), mTOR (1:1500), phospho mTOR (1:900), p62 (1:900), LC3 (1:800) and p70S6K (1:900) overnight at 4 °C. After incubation, the membranes were washed and incubated with horseradish peroxidase-conjugated secondary antibodies for 1 h at room temperature. The membranes are then washed and the bands were visualized using Enhanced Chemiluminescence Pico Kit (Thermo Fisher Scientific). The blots were stripped and re-probed for β-actin (1:5,000; monoclonal mouse; EMD Millipore, Billerica, MA, USA) as a loading control and densitometry analysis was done using “Image J” analysis software.

### 4.13. Immunohistochemistry Analysis

Cryoprotected animal brains were serially sectioned (20 µm) using a cryostat (Leica, Wetzlar, Germany) as described previously and the sections were washed twice with 0.01 M of PBS, pH 7.4. The sections were incubated for no longer than 10 min with 1% hydrogen peroxidase (in PBS) to inactivate tissue peroxidase. After two washes with PBS, the sections were blocked using blocking reagent (10% normal goat serum in PBS containing 0.3% Triton-X 100) for 30 min at RT and incubated with goat anti-rabbit tyrosine polyclonal antibody (1:1000) overnight at 4 °C. Sections were then rinsed twice with PBS and incubated with biotinylated secondary anti-rabbit (1:1000) antibody for 1 h at room temperature. After incubation, sections were developed using the avidin-biotin peroxidase complex system (ABC kit, Vectastain, CA, USA) followed by 3,3′ diaminobenzidine (DAB) to visualize and analyze TH immunoreactivity. Stained sections were then coverslipped using DPX mounting medium and the slides were viewed under a light microscope (Olympus, Hamburg, Germany).

### 4.14. Assessment of TH-Ir Dopaminergic Neurons and TH-Ir Dopamine Nerve Fibers Loss

To analyze the loss of TH immunoreactive (TH-ir) neurons in the SNc area, three different levels of the medial terminal nucleus region were counted, and the average value was represented as percentage of control [46]. Striatal fiber loss was analyzed by measuring the optical density of TH-ir dopaminergic fibers in the striatum using Image J software. The optical density of TH-ir fibers at three different fields from each section with equal area within the striatum was measured for each rat, and an average of three areas were calculated and depicted as percentage of control. The optical density of the overlying cortex was taken as background measure and subtracted from the value generated from the striatum. The counting of TH-ir neurons and optical density of the TH-ir fibers were carried out by an investigator blind to the experimental groups.

### 4.15. Protein Estimation

Protein concentration from each sample was quantified using Pierce BCA protein assay kit (Thermo Fisher Scientific) following the manufacturer’s instruction.

### 4.16. Statistics

Data were expressed as the mean value ± SEM. One-way analysis of variance followed by Tukey’s test was performed to calculate the statistical significance between various groups using SPSS 12 software. In all the experiments, *p* < 0.05 was considered statistically significant.

## 5. Conclusions

Together, our study demonstrated that Val protected dopaminergic neurons via suppressing rotenone induced oxidative stress by reverting antioxidant defense mechanisms and decreased neuroinflammation by suppressing production of pro-inflammatory factors. In addition, Val modulated autophagy pathway by preventing rotenone induced vacuole accumulation and enhanced lysosomal degradation of alpha-synuclein. Thus, Val could be further developed as therapeutic drug for PD.

## Abbreviations

PD	Parkinson’s disease
ROS	Reactive oxygen species
GFAP	Glial fibrillary acidic protein
Cox-2	Cyclooxygenase 2
iNOS	Inducible nitric oxide synthase
Rot	Rotenone
SOD	Superoxide dismutase
CAT	Catalase
TNF-α	Tumor necrosis factor-alpha
IL-1β	Interleukin-1β
Iba-1	Ionized calcium-binding adapter molecule 1
TH	Tyrosine hydrolase
LC3B	Microtubule-associated protein 1 light chain 3 beta
mTOR	Mammalian target of rapamycin
4E-BP1	Eukaryotic translation initiation factor 4E (eIF4E)-binding protein 1
